# Determinants of Smooth‐coated Otter ( *Lutrogale perspicillata* ) Latrine Occurrence and Diet in the Karnali River, Nepal

**DOI:** 10.1002/ece3.74322

**Published:** 2026-09-26

**Authors:** Ramesh Kathariya, Ramesh Prasad Sapkota, Rajesh Sada, Aashish Kapali, Maheshwor Kathariya, Kanchan Thapa

**Affiliations:** ^1^ Central Department of Environmental Science Tribhuvan University Kathmandu Nepal; ^2^ WWF Nepal Baluwatar, Kathmandu Nepal; ^3^ Faculty of Animal Science, Veterinary Science and Fisheries (FAVF) Agriculture and Forestry University Bharatpur Nepal

**Keywords:** cast net, hard component analysis, scent‐marking, spraint analysis

## Abstract

Smooth‐coated Otter ( *Lutrogale perspicillata* ) depends on riverine prey and bank habitat for foraging and scent‐marking, but evidence from the large, braided rivers in Nepal is limited. We assessed habitat correlates of latrine presence patterns and diet composition of Smooth‐coated Otter in the lower section (39 km) of the Karnali River. Latrine presence‐absence was surveyed across 70‐belt transects distributed along both banks of the river with 21.43% presence and modelled using generalised linear model (binomial) with river characteristics. Diet analysis was done through hard‐component analysis of 32 spraints and spraint placement was examined with measured distance from river and substrate type. The presence of latrine sites decreased with increasing proximity to settlement, river width and water velocity, and was lower at sites with a retaining wall. Spraint placement analysis showed that higher spraint counts were observed with increasing distance from river (*r* = 0.481, *p* = 0.0046) and the highest count on sand substrate. Diet from spraint analysis revealed dominance of fish species followed by crabs and birds. Fishing net was observed in 46.9% of analysed spraints, indicating frequent interaction with fishing gear. Our findings suggest that maintaining escape cover, limiting bank modification and reducing fishing gear exposure should be prioritised to improve habitat suitability and to reduce emerging threats in the Lower Karnali River.

## Graphical abstract

The study shows empirical evidence linking riverbank habitat features, scent‐marking behaviour (latrines) and prey use remains limited for large, braided river in Nepal. Our dataset combines (i) plot‐based latrine presence–absence modelling, (ii) spraint placement patterns relative to river edge and substrate and (iii) prey identification from spraints, allowing an integrated interpretation of marking‐site selection and trophic reliance in a river corridor.

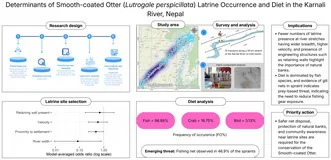

## 1 Introduction

Freshwater and wetland ecosystems occupy a small fraction of the Earth's surface yet support a disproportionately high share of global biodiversity and underpin key ecosystem services. These ecosystems, however, are among the world's most heavily affected ecosystems due to arrays of threats such as intensive water extraction, flow regulation, pollution, land‐use change, anthropogenic stress and climate change (Dudgeon et al. 2006; Vörösmarty et al. 2010; Xu et al. 2019).

Global assessments show that threats to riverine biodiversity are pervasive and often coincide with pressures on human water security, underscoring the need to address stressors at their source rather than relying only on costly remediation (Vörösmarty et al. 2010). For wetlands of international importance, major impact pathways commonly include pollution, biological resource use, natural system modification and agriculture and aquaculture (Xu et al. 2019). Climate change amplifies these pressures through altered hydrology and changes in ecosystem functioning, especially where water regimes are already disturbed (Moomaw et al. 2018; Strayer and Dudgeon 2010).

Across South Asia, these pressures have intensified in lowland floodplains where concrete infrastructure, riverbed sand and gravel extraction, and destructive fishing practices have altered habitats and depleted prey bases for aquatic predators (Khoo et al. 2021).

As an apex or a near‐apex predator, otters integrate changes in aquatic food webs and riparian environment, making them practical sentinels for tracking river health and evaluating management interventions (Gwachha et al. 2023). The Smooth‐coated Otter—SCO ( *Lutrogale perspicillata* ) is a social, group‐living mustelid strongly tied to lowland rivers and floodplains, but it also uses associated lentic habitats such as lakes, freshwater wetlands and oxbow lakes, and in some regions rice fields, mangroves and estuaries (Khoo et al. 2021; Raha and Hussain 2016). The species is assessed as Vulnerable on the IUCN Red List and Endangered on Nepal's National Red List of Mammals, with declines attributed to habitat loss and degradation, depletion of prey resources and direct persecution and illegal trade in parts of its range (Jnawali et al. 2011; Khoo et al. 2021). In Nepal, SCO are recorded from the major river systems, including the Karnali, but are sparsely distributed in the lowland Terai and remain poorly documented outside a few surveyed stretches (Acharya et al. 2023; Kathariya et al. 2023; Raha and Hussain 2016).

Diet is fundamental to understanding species' ecological roles, mapping trophic links, and interpreting energy and nutrient flows through ecosystems (Pineda‐Munoz and Alroy 2014; Ryol 2011). Dietary habits reflect phylogenetic history with some adaptations based on the environmental context, motivating documentation of diet of a particular species in spatial and temporal scales (Román‐Palacios et al. 2019). Diet information also informs conservation decisions, as it helps to identify key prey resources, periods of dietary bottlenecks and potential competition among sympatric predators (Cosby and Szykman Gunther 2021; Hoenig et al. 2022; Shehzad et al. 2012). In heavily modified landscapes, diet studies can further reveal how human activities reshape predator–prey interactions and alter the balance between wild prey and human‐associated foods (Dorresteijn et al. 2015; Shrestha et al. 2019; Van Scoyoc et al. 2023; Zhao et al. 2022; Zhong et al. 2022).

Dietary analysis involves determining and quantifying the species' food consumption pattern and characteristics providing the habits over the period (Bailey 2021). A wide range of methods is available for dietary analysis, each reflecting the scope of inference (Hoenig et al. 2022). Traditional approaches rely on direct observation, stomach contents or morphological identification of undigested remains in scats and pellets, while complementary tools such as stable isotopes and DNA‐based techniques extend temporal integration and improve taxonomic resolution (Hoenig et al. 2022; Pineda‐Munoz and Alroy 2014). Faecal DNA metabarcoding has become a widely used non‐invasive option for elusive species, but it carries known biases linked to sample quality, primer choice and reference databases (Ando et al. 2020; Shehzad et al. 2012).

For semi‐aquatic carnivores such as otters, non‐invasive diet assessment most commonly uses spraints, either through identification of hard remains (e.g., fish scales and bones, crustacean fragments) or, where feasible, through molecular techniques (Carss and Parkinson 1996; Shehzad et al. 2012). Hard‐remains analysis remains widely applied due to its low cost and suitability for large scale sampling. However, this method can underestimate the contribution of soft‐bodied or large prey and can occur detection biases that require cautious interpretation (Carss and Parkinson 1996; Cosby and Szykman Gunther 2021). In Nepal, policy, financial and resource constraints often limit otter diet studies to conventional spraint analysis rather than genetic methods, and research attention has tended to focus on flagship megafauna while small and semi‐aquatic mammals such as otters receive far less coverage (Kafle 2009; Lamichhane et al. 2019).

Because otters are elusive, indirect signs, particularly spraints and communal latrine sites, are widely used for surveys and monitoring (Moun et al. 2024; Oldham and Black 2009). Latrines are not random defecation points; they function as focal sites for olfactory communication and can convey information related to identity, territory, social status and recent use of resources (Kruuk 1992; Rostain et al. 2004). The location and intensity of sprainting can therefore respond to the abundance of prey species and to fine‐scale riverbank features (e.g., substrate, bank structure, cover) as well as broader landscape pressures such as disturbance and land use (Almeida et al. 2012; Moun et al. 2024). And the riverscape feature also represents the availability of prey species, thus also influencing behaviour including the latrine site selection of otters. Examining both latrine occurrence and diet composition within the same study system provides complementary insights into how habitat characteristics and food resources jointly influence the ecology of SCO. Assessing latrine site selection helps identify environmental factors associated with it; diet analysis provides information on the prey base supporting otter activity in those habitats.

Because latrines function as focal communication sites, we expected environmental correlates of latrine occurrence to differ from those associated with general otter habitat use (Kathariya et al. 2023). Specifically, we predicted that latrine occurrence would be greater at sites providing secure access to escape cover and lower human disturbance, while engineered riverbanks and other habitat modifications would negatively influence marking‐site selection.

The SCO's diet is predominantly fish, but varies with prey availability, season and site, and may include crustaceans, molluscs, insects, frogs, and snakes (Anoop and Hussain 2005; Basak et al. 2021; Baskaran et al. 2022; Jayasurya et al. 2023; Nawab and Hussain 2012b; Theng et al. 2016). Despite this general pattern, geographic and temporal coverage remain uneven, particularly in Nepal, limiting reference about context‐dependent feeding ecology. Given the variability in the SCO's diet due to factors like prey availability and stressors, it is imperative to gain comprehensive insights into the feeding ecology of this species for the conservation and management (Nawab and Hussain 2012b). Therefore, the proposed study combined analyses of latrine occurrence and the dietary composition ingested by SCO through hard remains analysis of undigested faecal matter commonly referred to as spraints. This traditional approach was used for the study because of the financial constraints to the expensive DNA metabarcoding.

## 2 Materials and Methods

### 2.1 Study Area

**FIGURE 1:**
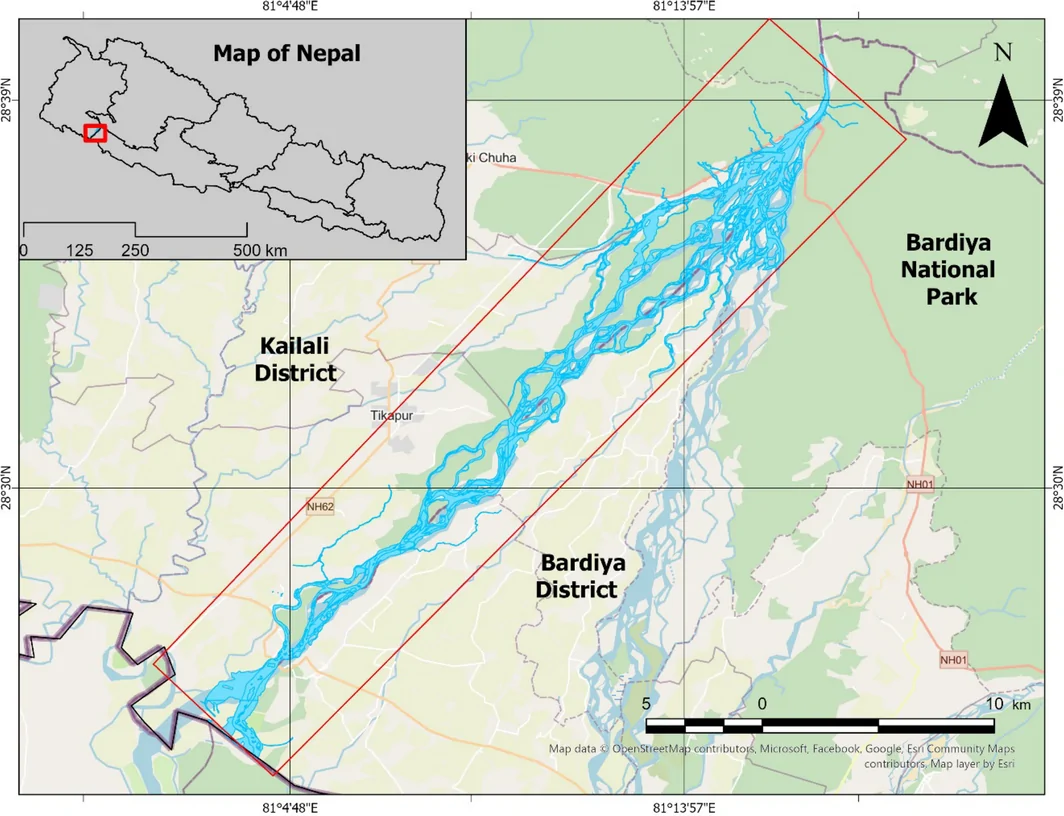
Map showing stretch in the Karnali River where the survey was done.

The research was conducted in the lower western branch (~39 km) of the Karnali River (Figure 1) below Chisapani Bridge, western Nepal which passes through part of a Terai floodplain mosaic of braided channels, sand bars, riverine forests and grasslands that support a suite of threatened freshwater fauna, including Gharial, Gangetic River Dolphin and SCO.

Fish species in the Karnali River include cyprinids such as *Barilius* spp. and *Schizothorax* spp., and migratory taxa such as *Tor* spp. and *Neolissochilus hexagonolepis* (Manandhar et al. 2023), providing an important prey base for semi‐aquatic predators.

### 2.2 Sampling and Environmental Variables

First, ethical approval from the Department of National Parks and Wildlife Conservation and the Bardiya National Park was received for field visit and sample collection from the western branch of lower Karnali River.

**FIGURE 2:**
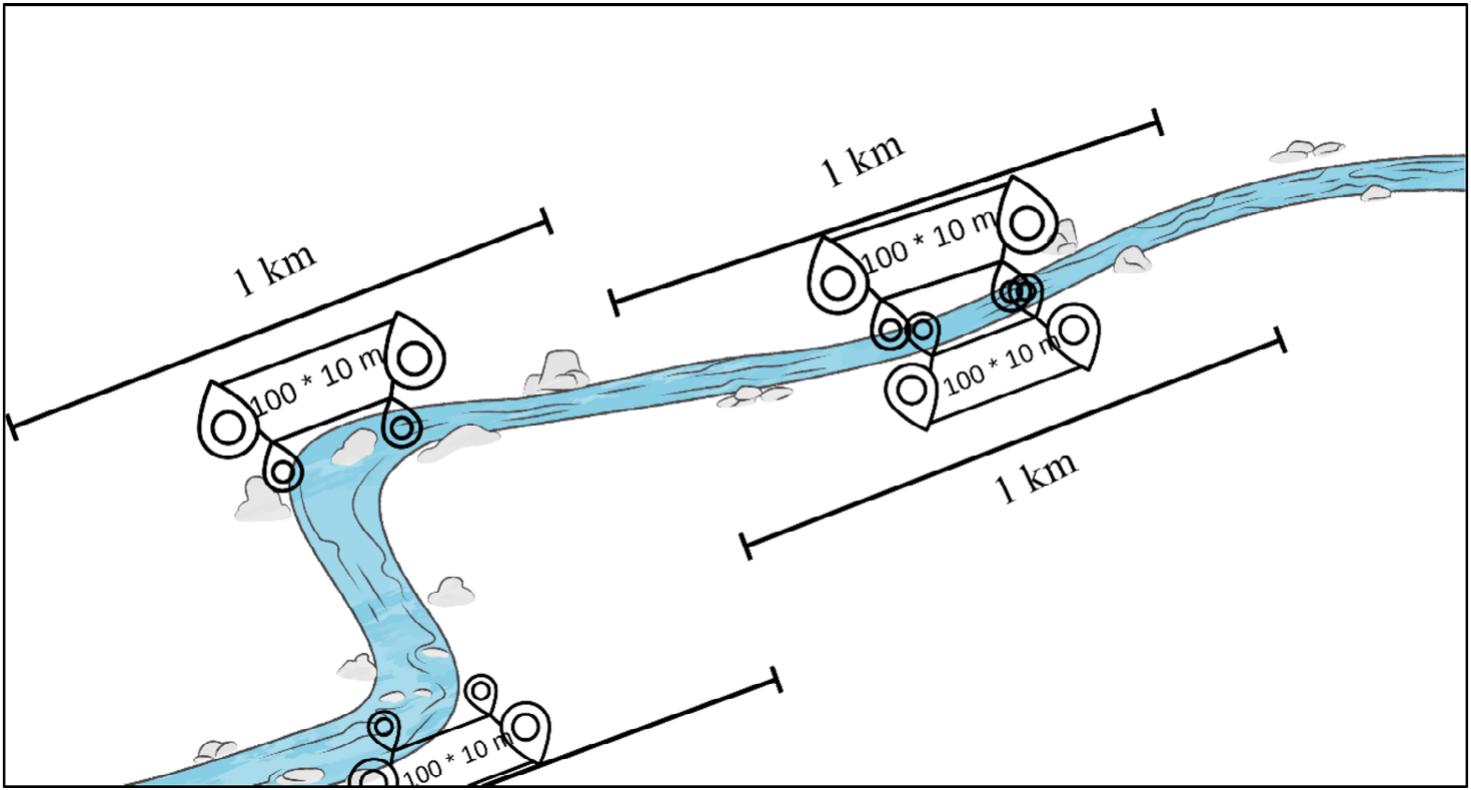
Survey design for latrine site selection and dietary analysis of Smooth‐coated Otter.

A preliminary survey was conducted in October 2023 to obtain general information about the study area and to plan the research design. Following the research design, an intensive field survey and sampling was conducted from 1 January 2024 to 22 February 2024. The survey was done before the onset of monsoon to prevent washout of indirect signs of otter due to rain and flood as suggested by Gupta et al. (2020). A belt transect survey was laid on both banks of the river stretch. The river was divided into consecutive 1‐km segments following the actual river course, and a temporary nested sampling plot (100 m × 10 m) was established at the midpoint of each segment on both banks (Figure 2) (Anoop and Hussain 2004; Hussain and Choudhury 1997; Nawab and Hussain 2012a).

Sites for latrine occurrence of the SCO were searched and noted, marking GPS location (Acharya and Lamsal 2010). The habitat characteristics, stream characteristics, disturbance intensity and types at each sample plot were noted on a printed record sheet as in Table 1 (Basak et al. 2021; Khan 2014).

**TABLE 1:** Habitat variables recorded in the field survey of SCO.

Variable	Data type	Description
Bank convexity	Continuous	Bank convexity was measured by using formula: Area of window (10 m × 100 m)/Total area of window considering the slope.
Elevation (m a.s.l.)	Continuous	Elevation at the plot was measured using the Garmin 64S.
Bank height (cm)	Continuous	Bank height was measured by using measuring tape.
Slope of riverbank (°)	Continuous	Slope of the river was measured by using clinometer.
River velocity (m/s)	Continuous	River velocity was measured by using floating method. In this method, we floated dry wooden stick for 100 m and recorded the time taken to pass 100 m. Then we used formula: velocity = distance in m/time taken to pass the distance (second).
Island (Braided bar)	Binary	Binary codes were used based on presence (1) and absence (0) of braided bar.
River width (m)	Continuous	Width of river from plot to braided bar or another bank was measured by using Nikon Forestry Pro Range Finder.
Retaining wall	Binary	Binary codes were used to record the data based on presence (1) and absence (0) of gabion wall on the plot.
Bank type	Categorical	Bank type was recorded based on field observation (Sand, Stone, Sand + Stone, Sand + Vegetation, Stone + Vegetation).
Land use	Categorical	Land use was recorded based on field observation (flood plain, agriculture, road, river construction).
Vegetation composition	Categorical	Name of plant species found in the sample plot based on field observation later verified by botanists.
Vegetation cover (%)	Continuous	Vegetation cover was estimated by ocular estimation method.
Escape distance (m)	Continuous	Escape distance was measured as distance of the plot to nearest gabion wall or vegetation area where SCO can hide to escape, measured by using Nikon Forestry Pro Range Finder or measuring tape.
Proximity to settlement (m)	Continuous	Proximity to settlement is distance (aerial) of plot to nearest settlement measured by using Google Earth Pro.
Disturbance intensity	Ordered	Disturbance intensity was measured based on field observation (High: sites of stone or sand extraction and less proximity distance; Moderate: sites of less human movement and high proximity distance: Low: sites of no or limited human movement and high proximity).
Disturbance type	Categorical	Disturbance type was noted based on field observation (Fishing, Human movement, River construction/Sand and gravel extraction).
Latrine site	Binary	Binary codes were used to record the data based on presence (1) and absence (0) of latrine site in plot and transect.

### 2.3 Fish Diversity and Reference Sample Preparation

Fish species richness was assessed by using the Cast Net method by throwing the cast net 50 times at each 5 km within a 500 m stretch with varied river characteristics (modified from IFC 2021). The cast net of mesh size = 5 mm, span diameter = 3.8 m and 2.6 kg was used to prepare a reference checklist of fish present in the Karnali River (Basak et al. 2021; IFC 2021). Thus, captured fishes were immobilised using clove oil, photographed, identified (local name and common name) and measured length (standard, total and fork). Identification of fishes was done with the help of local fisher folks (local names), using standard reference books (Shrestha 2008), and with confirmation from fish experts from Agriculture and Forestry University for common name (Baskaran et al. 2022).

The fish species found in the cast net was surveyed in the local market to get scales from five different parts (head, dorsum, underpart of pectoral fin, abdomen and tail) and was mounted for permanent slides using Dibutylphthalate Polystyrene Xylene (DPX) with a series of different concentrations of alcohol to identify fishes at genus level (Anoop and Hussain 2005; Wilson and Namaskari 2020).

The bone colour and structure, presence of exoskeleton, feathers and hairs were used to identify snakes, frogs, insects, crabs, birds and mammals to their lowest taxonomic rank (Anoop and Hussain 2005). Thus, prepared reference materials were used to identify taxa present in the spraint of SCO.

### 2.4 Spraints Collection and Processing

The spraints were collected along the river transect and kept in vials with 70% ethanol as preservative with a collection tag labelled with collection number, date, colour, shape, length, diameter and microhabitat in it (Basak et al. 2021; Hermsen and Maarseveen 2012). For each marking site, we recorded the substrate type (sand, stone, mould), distance of spraint from the river, and number of spraints. The detail also included collection date, spraint condition, colour, length, diameter and collection number. One communal sprainting event at one site was considered as one spraint within a 1 m radius (Anoop and Hussain 2005; Basak et al. 2021; Kruuk et al. 1986). The identification of the spraint was done by using de Silva (2011).

The old spraints (dry) were soaked in 0.7% of Na_2_CO_3_ (oxidising agent) to remove mucus and other decomposed matter (Basak et al. 2021). The spraint was washed individually under running tap water and with a sieve of size 0.5 and 1 mm (Anoop and Hussain 2005; Basak et al. 2021; Wilson and Namaskari 2020).

The undigested remains from spraint (scales, exoskeletons, bones, feathers) were mounted in slides with the same procedure as mentioned for reference slide preparation for fish scales and were identified to the lowest taxonomic level as possible using manuals and reference slides under binocular microscope (Hermsen and Maarseveen 2012).

### 2.5 Data Analysis

All statistical analyses, including both descriptive statistics and inferential testing, were conducted in R version 4.5.1 with RStudio IDE version 2025.9.1.401 (Cucumberleaf Sunflower) (R Core Team 2025). We first began with descriptive statistics of the results through using the power of central tendency using the *psych* package for distance to nearest water source and scat counts, and group‐wise summaries (Revelle 2025).

The number of spraints per location was discrete and rightly skewed and since, the data included only positive sites, the data count was zero‐truncated, we used Spearman's correlation to assess distance of spraint count with distance from active channel of river. Furthermore, we used the Kruskal‐Wallis rank‐sum test and further validated it with Dunn's pairwise comparison—Holm adjustment, to identify differences between substrate categories. All the statistical tests were evaluated at *α* = 0.5.

For the environmental predictors influencing the latrine site selection, we began by inspecting the dataset and converting categorical variables into factors to ensure proper handling in regression models. Missing values were removed using the na.omit() function to maintain consistency across analysis.

To reduce multicollinearity among predictors, we calculated variance inflation factors (VIF) using the *car* package (Fox and Weisberg 2019). Variables with VIF > 2.5 were excluded to improve model interpretability and coefficient stability (Johnston et al. 2018).

We first fitted a generalised linear model (GLM) with a binomial error distribution and logit link function to assess the influence of habitat variables on latrine presence. We further used dredge function in the *MuMIn* package to select best candidate models restricting to a maximum of three predictors to reduce model complexity (Bartoń 2010).

The candidate models were ranked using Akaike's information criterion corrected for small sample size (AICc). Models with ΔAICc ≤ 2 were considered to have comparable empirical support. While we had more than one candidate model being equally good, we used full model averaging and assigned a predictor a zero coefficient when absent in a model to provide a conservative estimate of predictor effect.

To determine relative occurrence or food preferability of SCO, frequency of occurrence (FO%) was determined after identifying species from spraint (Anoop and Hussain 2005; Fonseca da Silva et al. 2008; Wise et al. 1981).

## 3 Results

### 3.1 Spraint Location

A total of 70 transects (~39 km) along both banks of the Karnali River were surveyed. Latrine sites were recorded at 15 plots (21.43%), whereas 55 plots (78.57%) of 70 plots had no latrine evidence. This means roughly one transect with presence of the spraint was followed by three transects with absence. Spraint clustering was examined across 32 latrine sites (15 within the nested plot and remaining outside the nested plot). Distances from the active channel ranged from 13 to 1021 m. The median distance was 200 m, while the mean was higher (279.5 m). The interquartile range (Q1–Q3) was 140–343 m, so half of all locations fell within a ~203 m band around the mid‐range of distances.

**FIGURE 3:**
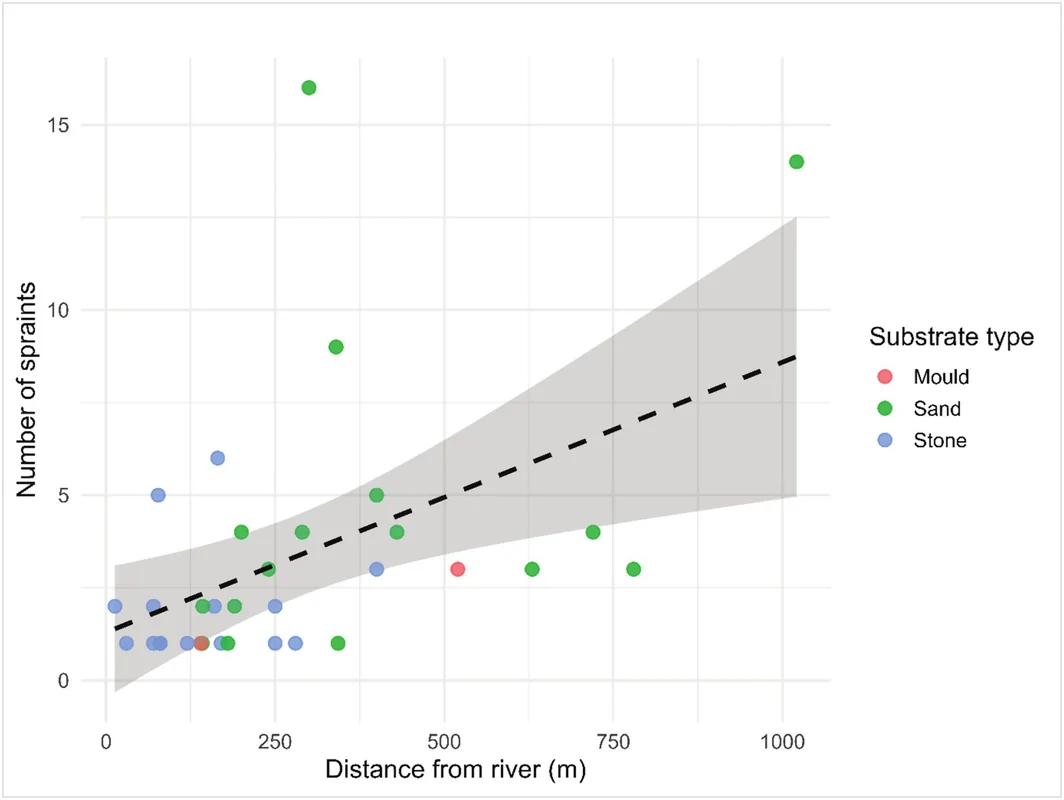
Correlating spraint count with distance from river (points coloured by substrate type; dashed line = linear fit).

Distance from the river was positively associated with spraint count (Spearman's *ρ* = 0.531, *p* = 0.0015). Thus, the location farther from the active river channel possesses a greater number of spraints (Figure 3).

**FIGURE 4:**
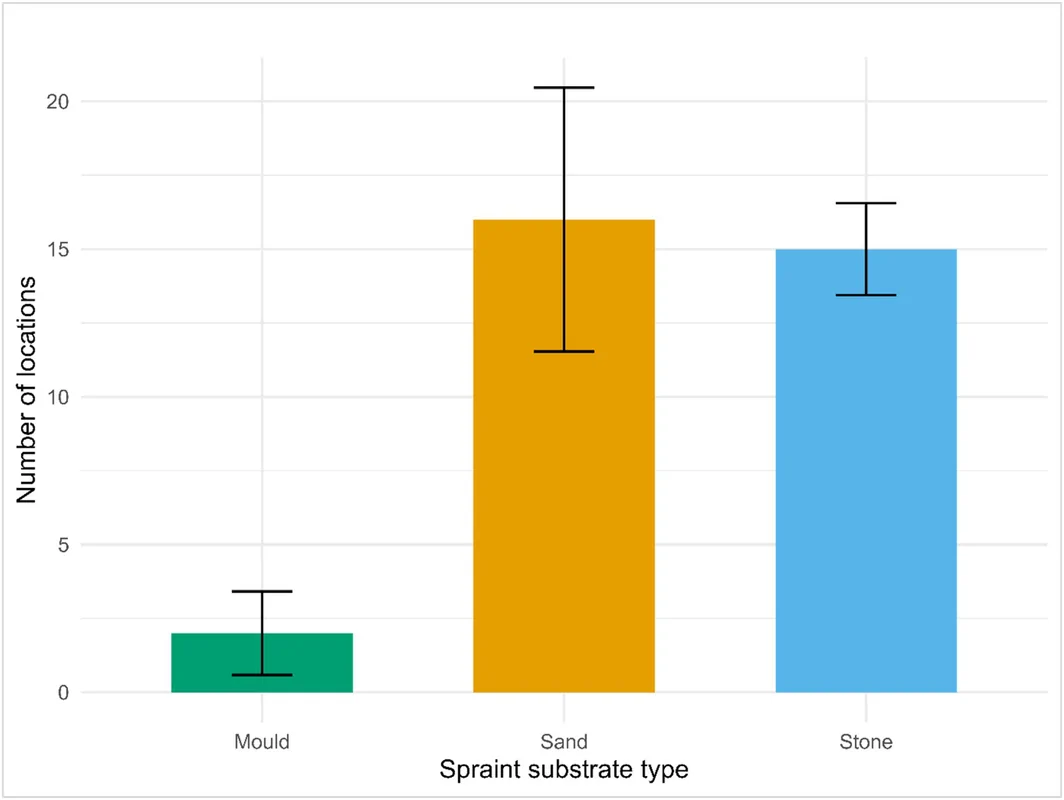
Number of locations by substrate type with error bars (± standard deviation of spraint counts).

We also summarised the substrate where spraint was found (mould, sand and stone). Sandy substrate supported highest count (4.75 ± 4.46; *n* = 16) and the highest variability (Figure 4). Stone and mould sites had lower mean counts (both means 2.00) and lower maxima (6 and 3). Spraint counts differed significantly among substrate types (Kruskal–Wallis *χ* ^2^ = 6.390, df = 2, *p* = 0.041). Dunn's pairwise comparison with Holm adjustment showed that spraint counts were significantly higher on sand than on stone substrate (*Z* = 2.477, adjusted *p* = 0.0398). However, the differences between mould and sand and between mould and stone were not statistically significant.

### 3.2 Latrine Site Selection

The predictor variables were screened at first for collinearity to increase the robustness of the model. The multicollinearity test with the VIF tool excluded four predictors (slope, convexity, proximity to road, total width of river, bank type, land use and substrate type) with VIF > 2.5, resulting in a final set of 10 predictors: elevation, distance to escape cover, proximity to settlement, bank height, river width, island presence, velocity, vegetation cover, disturbance intensity and retaining wall.

With binomial GLM, the six predictors showed a statistically negative association (significant) with latrine presence (Table 2). The occurrence of latrine sites decreased with increasing distance to escape cover, increasing distance from settlements, increasing river width and increasing flow velocity.

**TABLE 2:** Logistic regression coefficients for latrine presence.

Coefficients	Estimate	SE	*Z*‐statistic	*p*
Intercept	8.523	3.268	2.607	0.009**
Elevation	−0.007	0.016	−0.477	0.633
Escape distance	−0.016	0.006	−2.350	0.018*
Proximity to settlement	−0.002	0.000	−2.756	0.005**
Bank height	−0.002	0.003	−0.798	0.424
Width of river	−0.021	0.007	−2.929	0.003**
Island	−1.800	0.910	−1.977	0.048*
Velocity	−2.106	1.011	−2.082	0.037*
Vegetation cover	0.010	0.019	0.568	0.569
Disturbance intensity	−0.095	0.534	−0.177	0.858
Retaining wall	−2.311	0.892	−2.590	0.009**

*Note:* Signif. codes: 0 ‘***’ 0.001 ‘**’ 0.010 ‘*’ 0.050.

In addition, islands and retaining walls were associated with lower odds of latrine occurrence compared to sites without these features. The predictors elevation, bank height, vegetation cover and disturbance intensity showed no detectable effects in the multivariable model (Table 2).

Since the model with six predictors was too complex, we simplified the model complexity into a maximum of three predictor components. Out of 176 candidate binomial models we created using dredge function in the *MuMIn* package, a total of two models were retained with ΔAICc ≤ 2 (Table 3). Both models converged, and no complete or quasi‐complete separation was detected.

**TABLE 3:** Competitive candidate models explaining latrine occurrence in the Lower Karnali River.

Model	Predictors	*K*	log‐likelihood	AICc	ΔAICc	Weight
1	Distance to settlement + Velocity + River width	4	−42.778	94.010	0	0.711
2	Distance to settlement + Retaining wall + River width	4	−43.677	95.800	1.800	0.289

**FIGURE 5:**
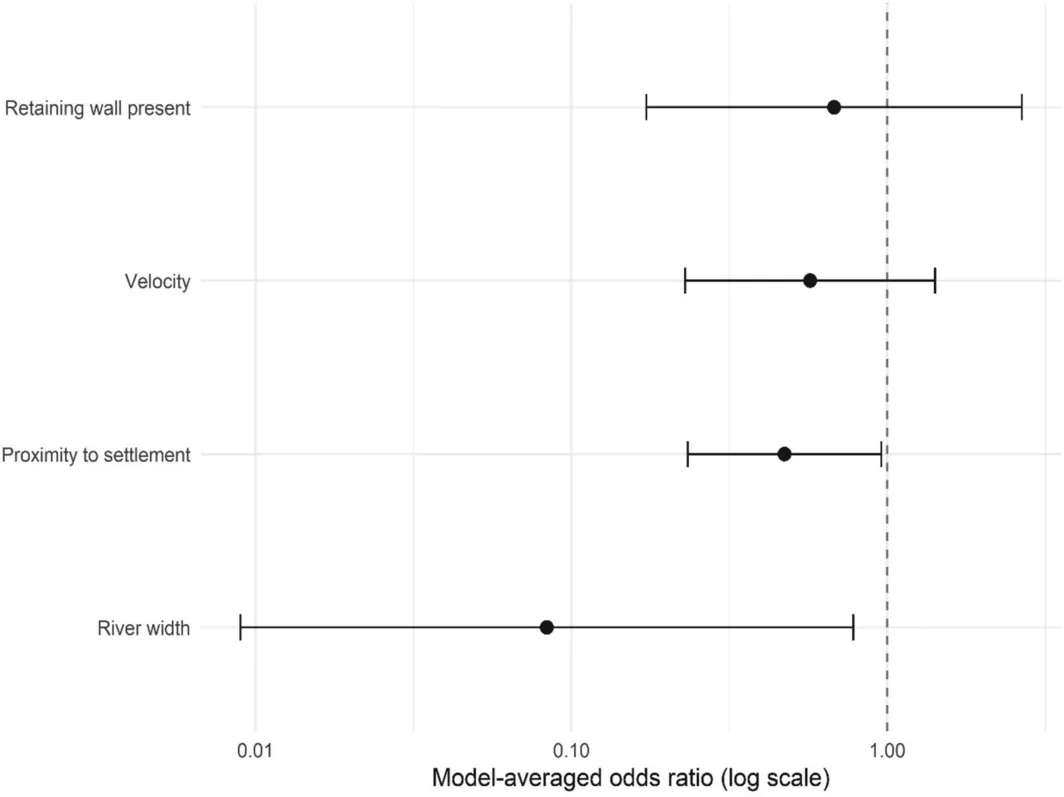
Full model‐averaged odds ratios and 95% confidence intervals for predictors represented in the competitive model set (ΔAICc ≤ 2). Continuous predictors were standardised, and their odds ratios represent a one‐standard‐deviation increase. The dashed line indicates no change in odds (OR = 1). Point shapes represent summed Akaike‐weight support.

Distance to settlement and river width occurred in both competitive models and each had a summed Akaike weight of 1.00 while full model averaging (Figure 5). The latrine occurrence declined with increasing distance from settlement. A one‐standard‐deviation increase in distance from settlements was associated with approximately 53% lower odds of latrine occurrence. River width also had a negative association with latrine occurrence. A one‐standard‐deviation increase in river width was therefore associated with approximately 92% lower odds of latrine occurrence. The water velocity and presence of a retaining wall occurred in only one of two models and both had negative full model averaged estimates.

### 3.3 Diet Composition

The fish sampling could capture ten fish species. Of these, five species with well‐developed scales ( *Badis badis* , *Channa marulius* , *Labeo dero* , *Pethia conchonius* and *Tor putitora* ) were processed for permanent slide preparation to facilitate the identification of fish scales recovered from the spraints of the SCO. Although *Macrognathus pancalus* and *Pseudambassis baculis* had scales, permanent slides were not prepared because their scales were too small and fragile for reliable processing. The scaleless species, *Acanthocobitis botia* , *Mystus bleekeri* and *Anguilla bengalensis* , were also excluded from slide preparation because they lack diagnostic scales.

**FIGURE 6:**
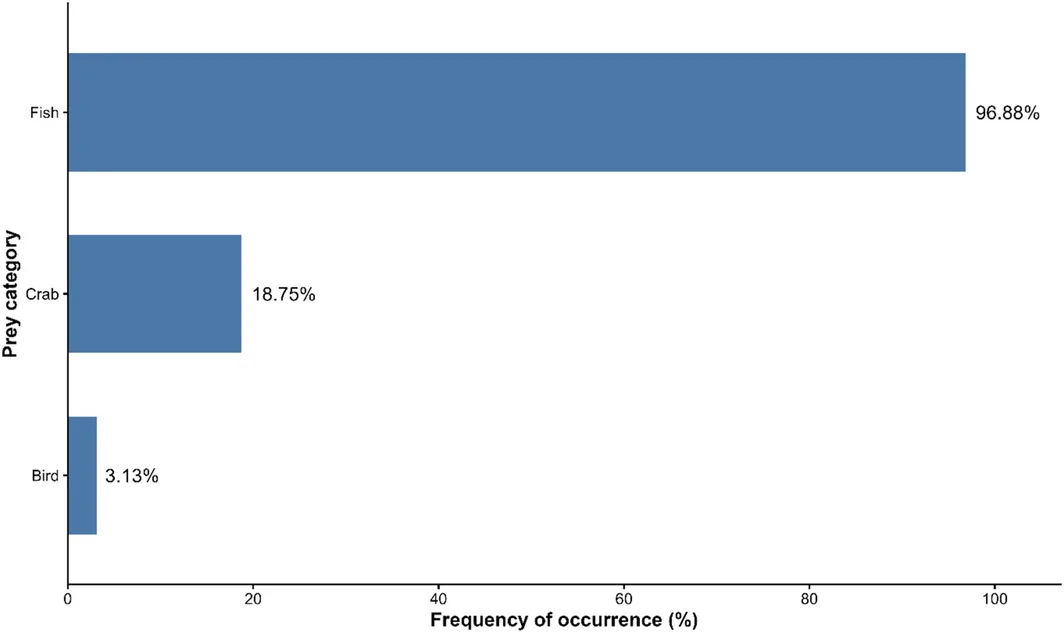
Frequency of occurrence of major prey categories detected in 32 SCO spraints.

The diet of SCO was assessed from 32 spraint samples using hard component analysis. The fish remains were almost universal (31/32; 96.88%), while crab remains were found in six samples (18.75%) and birds in one sample (3.13%) (Figure 6). Because a single spraint can contain multiple prey types, the FO values across categories do not sum to 100%. Within fish remains, *Labeo dero* was the most frequently observed taxon (21/32; 65.62%), followed by *Tor putitora* (10/32; 31.25%). A threatening case observed during the laboratory analysis was the presence of a fishing net wrapped in the spraints of the SCO (*n* = 15 out of 32 spraints).

## 4 Discussion

Our research provides evidence that latrine site occurrence of SCO in the Lower Karnali River is influenced by a combination of river geomorphology, hydrology, escape opportunities and human disturbance. This is the first study to systematically examine the latrine site occurrence in the stretch. The binomial GLM results indicated that escape distance, proximity to settlement, river width, presence of island, river velocity and presence of retaining wall were significantly associated with latrine occurrence (*p* < 0.05). But because of model complexity, we tried to reduce dimension with predictors no greater than 3 in a single model. The negative association of latrine sites with proximity to settlement coincides with the studies that demonstrates lower sprainting or reduced site use under gradient of anthropogenic disturbance (Awasthi et al. 2025; Moun et al. 2024). Our result suggests that latrine sites of SCO are more likely in relatively narrower and slower sections of the river. This pattern is consistent with the interpretation that narrower and stiller sections of the river provide more stable marking surfaces and predictable access points. A similar negative association between the river width and occupancy signs has already been documented along the same stretch of the Karnali River, indicating that this response may be persistent with latrine site selection as well in the Lower Karnali River system (Kathariya et al. 2023). In Shuklaphanta National Park, the presence of otter increased with wider water channels, suggesting width can act as a proxy for prey base and habitat quality depending on the local geomorphic and disturbance context (Awasthi et al. 2025). The presence of retaining wall has a strong negative relation with latrine site occurrence. This indicates that the engineered banks reduce suitable marking and grooming surface and possibly restrict access and den opportunities. In Cauvery River system, the latrine sites were characterised by lose sand and rock, hard‐packed sand, stone, vegetation and canopy cover were less represented at otter preferred sites, which suggests physical characteristics of bank surface is critical for grooming and sprainting (Shenoy et al. 2006). Also, riverbank concretisation and habitat modification have been highlighted as threat to habitat suitability for otters in human‐dominated landscapes (Jayasurya et al. 2023).

The count for spraint (a communal latrine site) ranged from 1 to 16 (mean = 3.33 and median = 2), which indicates that the most communal latrine sites were of low use. The spraint count increased with distance from the edge of the river, suggesting marking sites may occur where spraints are less likely to be washed away by the water shore or daily fluctuation of water.

The analysis for substrate where spraint was found showed sand to have a higher count and variability than stone and mould. Sandy substrate provides a better grooming surface for SCO and increases detectability of spraints. However, mould did not differ significantly from either sand or stone. The result should therefore be interpreted specifically as evidence of greater spraint deposition on sand relative to stone, rather than as a complete preference ranking among all three substrate types. In the Cauvery River, loose sand was a defining feature of otter sites for grooming and territorial marking (Shenoy et al. 2006).

The higher frequency of fish occurrence in spraint of SCO coincides with multiple studies that reports fish as principal diet (exceeding 80% by FO and/or score bulk estimate) of SCO (Anoop and Hussain 2005; Hussain and Choudhury 1997; Theng et al. 2016). However, fish dominance is not consistent across all ecosystems and studies. In Kollidam and Thenpennai Rivers, fish represented 46%–53% of diet, and in a mangrove habitat fish comprised of 44% and crab 43%, indicating crab and other taxa can be a substantial prey in other ecosystems (Jayasurya et al. 2023; Wilson and Namaskari 2020). Similarly, an analysis in Goa found fish to be dominant but also reported substantial shrimp contribution, highlighting that coastal ecosystem can shift balance among aquatic prey (Dias et al. 2025). Our laboratory analysis also found minor count of bird remains and other studies has also reported occasional bird component. The presence of bird remain in our laboratory analysis indicates SCO opportunistic feeding behaviour beyond fish. Other studies also report occasional bird consumption which aligns with our study (Anoop and Hussain 2005; Theng et al. 2016). A total of four fish taxa were recorded in the spraint analysis. Two were identified as *Tor putitora* (FO = 31.25%) and *Lebeo dero* (FO = 65.62%), while two did not matched any reference scale collected from the river and were reported as unidentified 1 (FO = 3.125%) and unidentified 2 (FO = 9.38%). The two species unidentified must be fish species other than ten species recorded in the survey, The identified fishes with high FO in otter spraints are local fishes in the area that are utilised by the local communities for their subsistence. Such overlap can shape negative perceptions when fisheries decline or when there is fishing net damage (Dias et al. 2025; Trivedi and Variya 2023).

The presence of gill net fragments in otter spraint provides direct evidence that otters are frequently interacting with fishing gear in Lower Karnali River. In addition, abandoned or left fishing gears are a wildlife hazard because they entangle and injure the animals. A possible pathway in our study is likely due to ingestion while removing or consuming fish from gill nets. SCO has been reported to take fish directly from gill nets and to damage gears (Trivedi and Variya 2023). Thus, it is crucial to consider the net in spraint as a conflict as well as a conservation hazard that needs timely intervention.

In the Lower Karnali River, recently, Rajapur and Tikapur municipalities have jointly declared Nepal's first Fish Sanctuary (3.9 km^2^) in the same stretch to this research (WWF Nepal 2026). This declaration is a practical pathway for conservation by improving prey population and regulating fishing pressure in the area.

The latrine occurrence and dietary analysis provide complementary evidence of how SCO uses the river stretch. Latrine sites closer to escape cover may facilitate safer movement between marking sites and foraging habitats, while narrower river sections may provide easier access between the channel and riverbank. The high occurrence of fish in spraints further confirms the importance of riverine prey resources in supporting otter activity. However, the occurrence of fish species in spraints cannot be directly linked with the characteristics of latrine sites because fish abundance and microhabitat use were not measured. Therefore, our results show a connection between suitable marking habitat and the use of riverine fish species, but not a direct spatial association between individual latrines and the capture locations of identified prey.

## 5 Conclusion

This study provides a baseline for the prey‐base conservation of SCO in Nepal. It lays site‐level evidence on how SCO use the lower section of the Karnali River for latrine marking, and how this links with prey use and human pressure. Our analysis suggests latrine site occurrence was concentrated in comparatively narrower and faster sections of the river stretch and was also associated with distance to settlements and presence of retaining wall. This suggests channel instability and riverbank infrastructure reduce the suitability of repeated marking. The occurrence of latrine sites closer to settlements suggests that marking activity overlaps with river sections that are likely to experience fishing, movement of people, livestock use, riverbank modification and other human activities. This relationship should not be interpreted as preference for human disturbance, but it indicates that otter conservation cannot be limited to remote or apparently undisturbed river sections. This overlap also suggests potential conflict between human and otter.

The spraint placement pattern indicated marking intensity was concentrated at a small number of locations. The spraint counts in a latrine site increased with distance from the river and were highest on sand substrate. The sand substrate provides a better grooming surface and improves marking persistence to SCO. Dietary analysis confirmed SCO's major preference as fish followed by crabs and birds occurring occasionally. The dominant fish taxa identified ( *Labeo dero* and *Tor putitora* ) indicate strong dependence on riverine fish resources which are equally important to the local communities living there. A key concern emerging from this study was the frequent occurrence of fish net material wrapped in spraints. These findings indicate a need for practical action to reduce destructive fishing, and safer disposal of damaged nets through community awareness. These measures should be implemented through local governments in collaboration with local community‐based organisations and river‐dependent Tharu and Sonaha communities.

## Author Contributions

**Ramesh Kathariya:** conceptualization (equal), data curation (equal), formal analysis (equal), investigation (equal), methodology (equal), writing – original draft (equal), writing – review and editing (equal). **Ramesh Prasad Sapkota:** conceptualization (equal), data curation (equal), formal analysis (equal), methodology (equal), project administration (equal), software (equal), supervision (equal), writing – review and editing (equal). **Rajesh Sada:** conceptualization (equal), funding acquisition (equal), supervision (equal), writing – review and editing (equal). **Aashish Kapali:** investigation (equal), project administration (equal), writing – review and editing (equal). **Maheshwor Kathariya:** data curation (equal), investigation (equal), writing – review and editing (equal). **Kanchan Thapa:** supervision (equal), writing – review and editing (equal).

## Funding

This work was supported by WWF Nepal, WWF UK and UK Darwin Initiative (30‐017).

## Disclosure

All authors contributed to the research design, methodology and writing of the manuscript, and approved the final version for submission.

## Conflicts of Interest

The authors declare no conflicts of interest.

## Supporting information


**Data S1:** ece374322‐sup‐0001‐Data_Otter.xlsx.

## Data Availability

Data available in article Supporting Information.
